# Molecular mechanisms of heptaplatin effective against cisplatin-resistant cancer cell lines: less involvement of metallothionein

**DOI:** 10.1186/1475-2867-4-6

**Published:** 2004-10-19

**Authors:** Cheol-Hee Choi, Yoon-Jung Cha, Chun-San An, Kyung-Jong Kim, Kweon-Cheon Kim, Sung-Pyo Moon, Zang Hee Lee, Young-Don Min

**Affiliations:** 1 Research Center for Resistant Cells, Department of Pharmacology, Chosun University Medical School, 375 Seosuk-dong, Dong-gu, Gwangju 501-759, South Korea; 2 Department of Surgery, Chosun University Medical School, 375 Seosuk-dong, Dong-gu, Gwangju 501-759, South Korea; 3 Department of Cell and Developmental Biology, College of Dentistry, Seoul National University, 110-747 South Korea

**Keywords:** Gastric cancer, heptaplatin, cisplatin, carboplatin, metallothionein

## Abstract

**Background:**

Heptaplatin is a new platinum derivative with anticancer activity against various cancer cell lines, including cisplatin-resistant cancer cell lines (Cancer Chemother Pharmacol 1995; 35: 441).

**Methods:**

Molecular mechanisms of heptaplatin effective against cisplatin-resistant cancer cell lines has been investigated in connection with metallothionein (MT). Cytotoxicity was determined by an MTT assay. MT mRNA, was determined by RT-PCR assay. Transfection study was carried out to examine the function of MT.

**Results:**

Of various gastric cancer cell lines, SNU-638 and SNU-601 showed the highest and lowest levels of MT mRNA, respectively, showing 80-fold difference. The IC_50 _values of SNU-638 to cisplatin, carboplatin and heptaplatin were 11.2-fold, 5.1-fold and 2.0-fold greater than those of SNU-601, respectively. Heptaplatin was more effective against cisplatin-resistant and MT-transfected gastric cancer sublines than cisplatin or carboplatin was. In addition, heptaplatin attenuated cadmium, but not zinc, induction of MT.

**Conclusion:**

These results indicate that molecular mechanisms of heptaplatin effective against cisplatin-resistant gastric cancer sublines is at least in part due to the less involvement of MT in heptaplatin resistance as well as its attenuation of MT induction.

## Introduction

Gastric cancer is the most frequently diagnosed and the second leading cause of cancer-related death in Korea. For many years, a few single agents such as 5-fluorouracil, doxorubicin, mitomycin C, and nitrosourea, have been considered to have significant antitumor activity in gastric cancer patients [1]. However, the response rate has been <30% and complete remission has been rare. Several combination chemotherapy regimens such as FAM (5-fluorouracil, adriamycin, and mitomycin C) have been attempted in order to improve the treatment outcomes. In a nonrandomized Phase II study for advanced gastric cancer, the FAM regimen achieved an objective partial response rate of 42% [2]. Some cisplatin-based combination chemotherapy regimens have shown high response rates [3,4].

**Figure 1:**
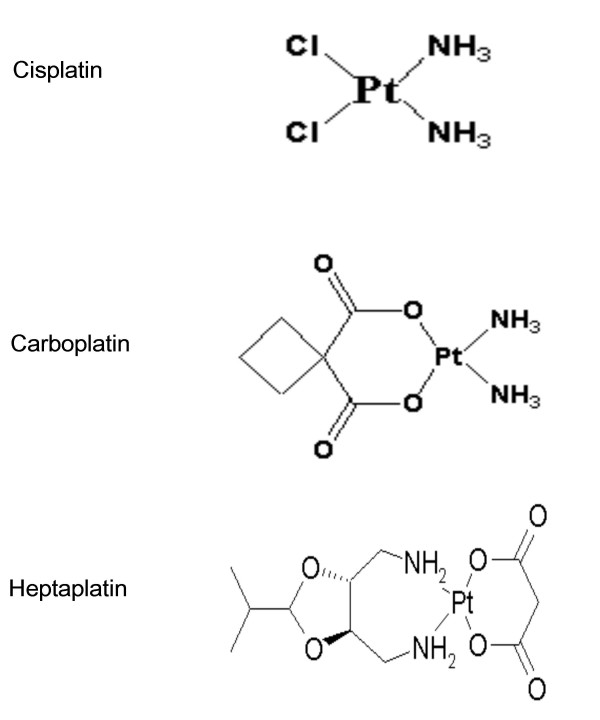
Structure of cisplatin analogs.

Despite the efficacy of cisplatin against gastric cancer, there were two major problems with this agent. Firstly, there are significant side effects, such as severe nausea and vomiting, nephrotoxicity, and neurotoxicity [5]. Secondly, the cancer cells show a primary or acquired resistance to cisplatin [6]. Therefore, extensive effort to develop novel cisplatin analogs with equivalent or greater antitumor activity and a lower toxicity has been made. Among them, carboplatin has reduced renal and gastrointestinal side effects than cisplatin [7]. However, carboplatin has no enhanced therapeutic efficacy over cisplatin and has not circumvented the acquired resistance to cisplatin. Therefore, it is essential to develop a new platinum drug with a greater potent antitumor activity as well as being effective against resistant cells. Recently, SK Pharmaceutical (Seoul, Korea) developed many cisplatin analogs, including heptaplatin (SKI-2053R) (Fig. 1). Heptaplatin exhibited a greater antitumor activity against a number of human cancer cell lines including gastric cancer as well as a lower nephrotoxicity [8,9]. In addition, heptaplatin 2053R is also effective against cisplatin-resistant L1210 leukemia cells (L1210-CPR) [10].

Metallothioneins (MT) are a family of stress-induced proteins with a wide variety of physiological functions, including protection against metal toxicity and oxidants. They may also assist in regulating cellular proliferation, apoptosis, and malignant progression. MT has a high affinity for heavy metals since it contains many cystein residues, which account for approximately 30% of the total amino acids in this protein molecule [11]. MT is a low molecular weight metal binding protein whose synthesis is induced by heavy metals, glucocorticoids and other factors [12]. Although the physiological role of MT is unclear, MT participates in detoxifying heavy metals or maintaining zinc and copper homeostasis [12]. Some reports have shown that MT has free radical scavenging ability *in vitro *[13,14] and MT expression also increases the cellular resistance to radiation damage [15]. The cells transfected with the bovine papilloma virus expression vectors containing the DNA encoding human metallothionein-IIA were resistant to cisplatin, melphalan, and chlorambucil but not to 5-fluorouracil or vincristine [16].

In this study, the effect of MT on the cisplatin analog-induced cytotoxicity was investigated in the gastric cancer cell lines. Since heptaplatin is less associated with MT, it is believed to be effective against cisplatin-resistant cells related to the high level of MT.

## Results

MT cDNA, whose expression level was higher than the wild-type AML-2, were isolated from the paraquat-resistant AML-2 cells [17,18]. The nucleotide sequence of the clone, containing an almost full-length cDNA copy of the human MT mRNA, was determined. Molecular cloning and nucleotide sequence analysis revealed that the hMT transcript is a novel hMT-I isoform, which was designated hMT-Ip, and exhibits a 98.4% homology with the hMT-Ie isoform, whereas it shows 86.9% homology with hMT-II, 83.8% homology with hMT-III and 62.9% homology with hMT-IV [18].

### MT mRNA expression in the gastric cancer cell lines

**Figure 2:**
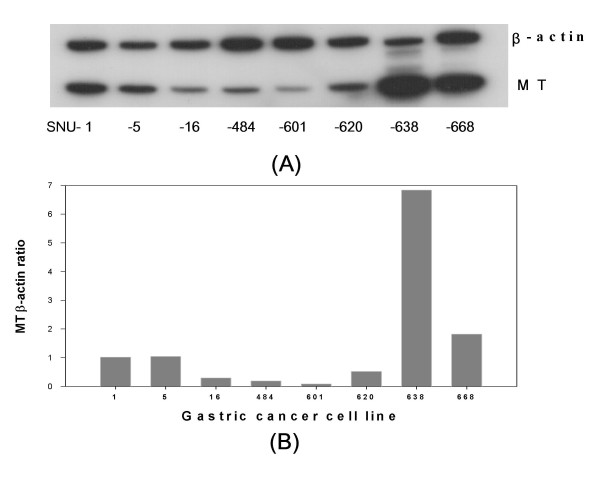
(A) MT mRNA expression in the gastric cancer cell lines. The expression level was determined by RT-PCR assay. β-actin was used as a control for RNA. The cDNA reverse-transcribed from the mRNA was individually amplified with each primer pair for the *MT *and β-*actin *genes. Aliquots of each PCR reaction mixture were separated on 7% polyacrylamide gel in TAE. The gel was dried and exposed on X-ray film overnight. (B) The ratio of MT/β-actin of SNU cell lines.

Using the RT-PCR assay, the MT mRNA was determined in the 8 human gastric cancer cell lines, SNU-1, SNU-5, SNU-16, SNU-484, SNU-601, SNU-620, SNU-638, and SNU-668. The gastric cancer cell lines showed differential expression level of MT mRNA, in which the SNU-601 cells expressed the lowest MT mRNA level whereas SNU-638 expressed the highest level (Fig. 2). The MT mRNA level of SNU-638 was 80 times higher than that of SNU-601.

### Comparative sensitivity of the gastric cancer cells that express MT differentially to cisplatin analogs

**Figure 3:**
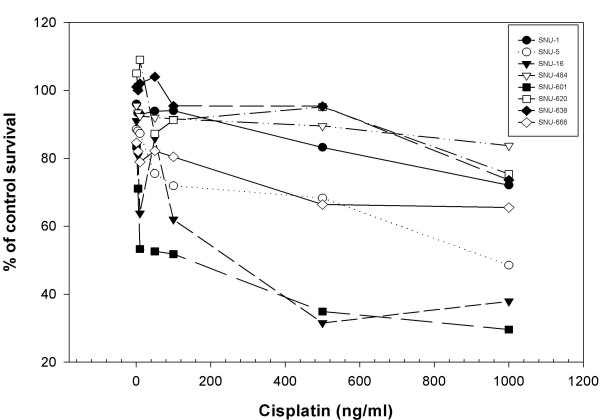
Cytotoxic effect of cisplatin in the gastric cancer cell lines. Cisplatin cytotoxicity was determined using an MTT assay.

The resistance of the gastric cancer cells that express MT to cisplatin was compared. SNU-16 and SNU-601, which showed the lowest MT expression levels, were sensitive but SNU-638, which exhibited the highest MT expression levels, were resistant to cisplatin (Fig. 3). On the other hand, the gastric cancer cell lines expressing moderate MT expression levels (SNU-16, SNU-484, SNU-620, SNU-5 and SNU-668) showed a lower correlation between the sensitivity to cisplatin and MT expression (Fig. 3). In addition, 3 gastric cancer cell lines with very low (SNU-484) or moderate MT levels (SNU-1 and SNU-620) which are as resistant to cisplatin as SNU-638 (Fig. 2 and 3), suggesting no involvement at all of MT in cisplatin resistance or the involvement of other resistance mechanisms except MT.

### Correlation of MT mRNA expression and sensitivity to the cisplatin analogs

**Figure 4:**
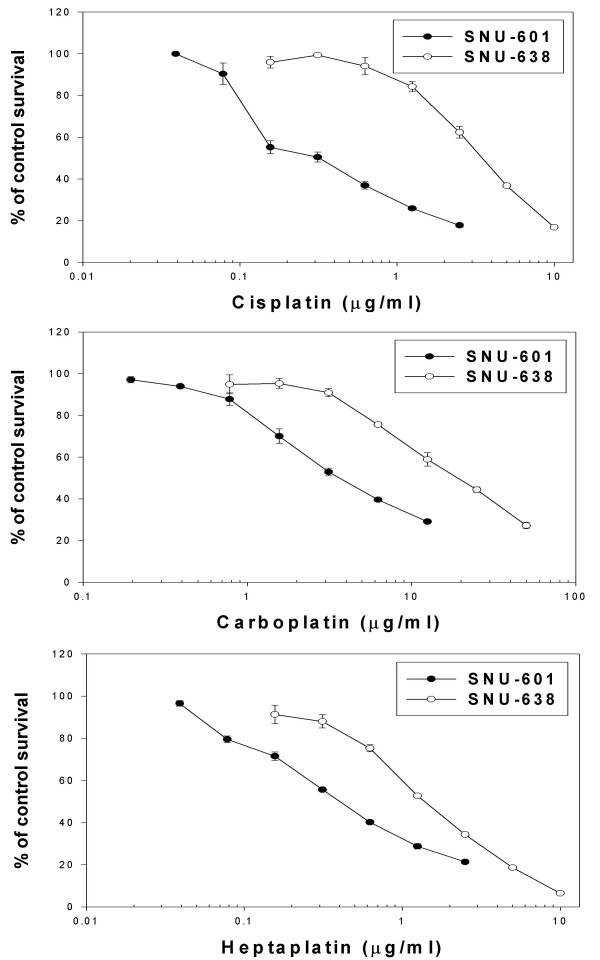
Cytotoxic profiles of the cisplatin analogs in the SNU-601 and SNU-638 cell lines showing low and high MT expression levels, respectively.

The cytotoxicity of the cisplatin analogs in the SNU-638 and SNU-601 cells, which expressed different MT levels, was determined by an MTT assay. The SNU-638 cells were approximately 11 times, 4 times and 2 times more resistant to cisplatin, carboplatin and heptaplatin, respectively, than the SNU-601 cells (Fig. 4 and Table 1), suggesting a lower involvement of MT in the resistance to heptaplatin.

**Table 1:** Comparison of cytotoxicities of the cisplatin analogs in various gastric cancer cell lines

	**Ratio of IC_50 _**^a^		
	
**Cell line**	**Cisplatin**	**Carboplatin**	**Heptaplatin**
638/601^b^	11.23	5.10	2.00*
CIS/601^c^	50.30	55.95	28.36*
MT/Mock^d^	54.77^e^	3.00	1.57*

^a^, The 50% inhibitory concentration (IC_50_) for a particular agent was defined as the drug concentration which results in a 50% reduction in cell number to the untreated control. IC_50 _values were determined directly from semilogarithmic dose-response curves. The means were obtained from the experiments carried out at least in triplicate.

^b^, The ratios of IC_50_values of SNU-638 to that of SNU-601 (Fig. 4)

^c^, The ratios of IC_50 _values of SNU-601/Cis to that of SNU-601 (Fig. 5)

^d^, The ratios of IC_50 _values of SNU-601/MT to that of SNU-601/Mock (Fig. 7)

^e^, The ratio calculated from extrapolated data obtained in SNU-601/MT (Fig. 7) *, P < 0.05 versus cisplatin or carboplatin group

### Comparison of resistance to cisplatin analogs in gastric cancer sublines resistant to cisplatin

**Figure 5:**
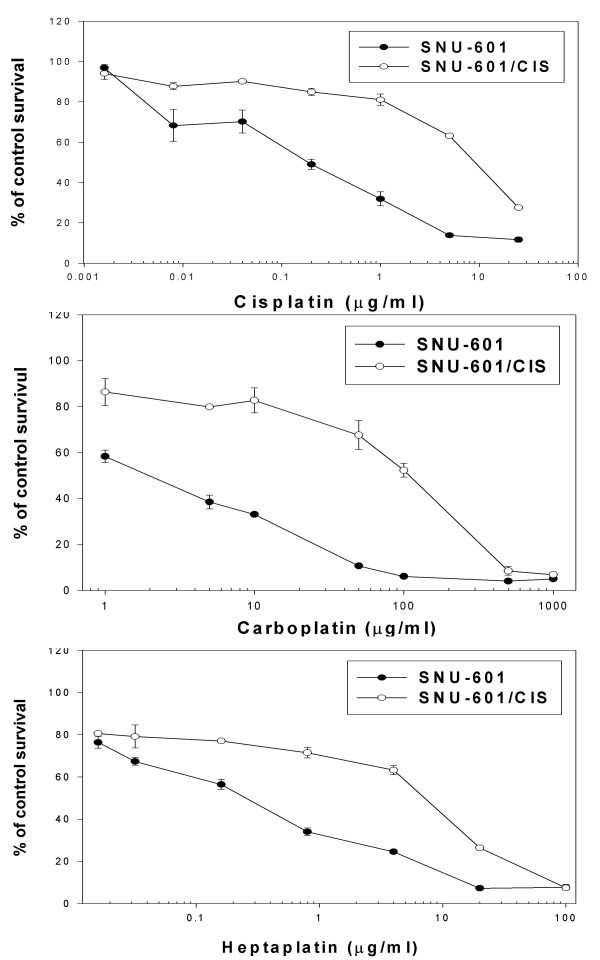
Cytotoxic effects of cisplatin, carboplatin and heptaplatin in the SNU-601/WT cells and its cisplatin-resistant subline SNU-601/CIS. The cytotoxicity was determined using the MTT assay. Mean ± SE of triplicate determination is given.

The cisplatin-resistant gastric cancer subline, SNU-601/Cis was selected in the presence of 2 μg/ml cisplatin. The SNU-601/Cis cells showed an approximately 49-fold, 56-fold and 29-fold increased resistance to cisplatin, carboplatin and heptaplatin, respectively (Fig. 5 and Table 1).

### Sensitivity of MT-transfected SNU-601 cells to cisplatin analogs

Sensitivity to cisplatin analogs was compared between SNU-601 transfected with the vector containing the full-length MT cDNA (pIRESneo2/MT) and with the empty vector (pIRESneo2/Mock).

**Figure 6:**
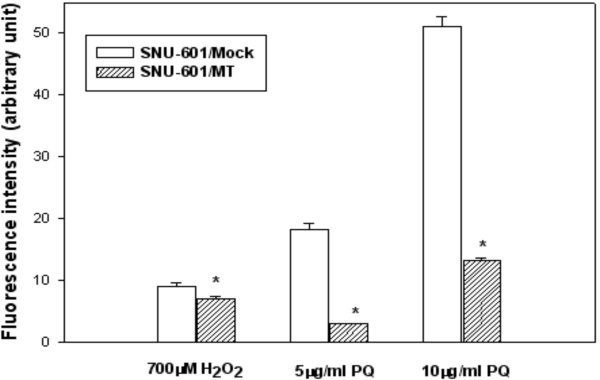
Comparison of ROS-scavenging activities between the SNU-601/Mock and the SNU-601/MT sublines. The reaction took place with 1 × 10^5 ^cells and 1 μM 2',7'-DCFH diacetate in 3 ml phosphate-buffered saline. The cells were exposed to H_2_O_2 _and paraquat for 4 hours. The fluorescence intensity was determined using a fluorometer with excitation wavelength at 485 and emission wavelength at 530 nm. The results are expressed as means ± SE (n = 3). PQ, paraquat; *, P < 0.05.

Although the SNU-601/MT showed a 66% increase in MT expression when compared to the SNU-601/Mock cells, the SNU-601/MT cells scavenged significantly ROS generated by hydrogen peroxide or paraquat as compared with those in SNU-601/Mock using a fluorescence probe (Fig. 6). This result suggests that SNU-601/MT expresses not only a higher quantity of MT than does SNU-601/Mock but also the MT functions to scavenge ROS.

**Figure 7:**
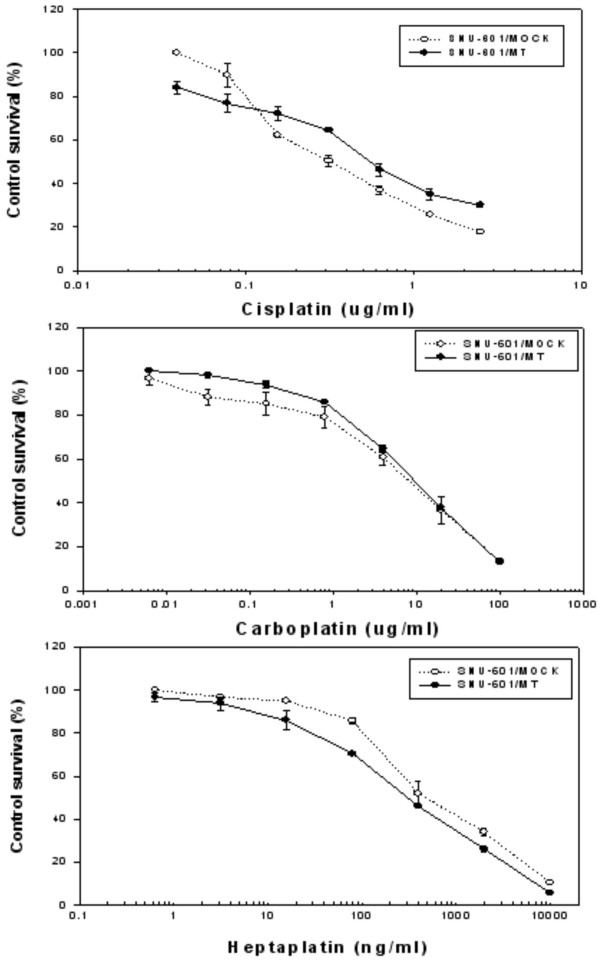
Cytotoxic effects of cisplatin, carboplatin and heptaplatin in the SNU-601/Mock and SNU-601/MT sublines. Cytotoxicity was determined using the MTT assay. Mean ± SE of triplicate determination is given.

The sensitivities of the SNU-601/MT cells to cisplatin analogs were tested using the MTT assay. The SNU-601/MT cells showed a lower degree of resistance to cisplatin and carboplatin, but was paradoxically sensitive to heptaplatin when compared to the SNU-601/Mock cells (Fig. 7).

### Comparison of MT mRNA expression in SNU-601 after treatment of zinc and cisplatin analogs

**Figure 8:**
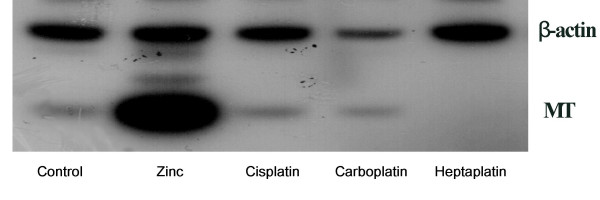
MT expression in the SNU-601 cell line after a treatment with zinc and the cisplatin analogs. The SNU-601 cells were treated with 100 μM ZnCl_2_, 0.6 μg/ml cisplatin, 2 μg/ml carboplatin, 0.2 μg/ml heptaplatin for 24 hrs. The MT mRNA expression level was determined by the RT-PCR assay.

To examine the effect of zinc and cisplatin analogs on MT mRNA expression, the maximum non-cytotoxic concentrations of each drug in the 3-day MTT assay were chosen and then treated for 1 day. After the treatment with 100 μM ZnCl_2_, 0.6 μg/ml cisplatin, 2 μg/ml carboplatin and 0.2 μg/ml heptaplatin for 24 hr, MT mRNA level was determined using the RT-PCR assay. The MT mRNA expression level was significantly increased by ZnCl_2 _but was decreased by heptaplatin. In contrast, there were no changes in MT mRNA expression level by cisplatin and carboplatin (Fig. 8).

### Effect of heptaplatin on heavy metal-induced MT expression in SNU-601 cells

Of the cisplatin analogs, heptaplatin is not only relatively less related to MT but also decreases the MT expression level. We tested whether or not heptaplatin could influence the heavy metal-induced MT expression. Each IC_50 _concentration of CdCl_2 _or ZnCl_2 _obtained from the 3-day MTT that did not influence the growth of the SNU-601 cells for 24 hr were used in this study. The SNU-601 cells were treated with 32 μM CdCl_2 _or 200 μM ZnCl_2 _in the presence or absence of 200 ng/ml heptaplatin for 24 hr.

**Figure 9:**
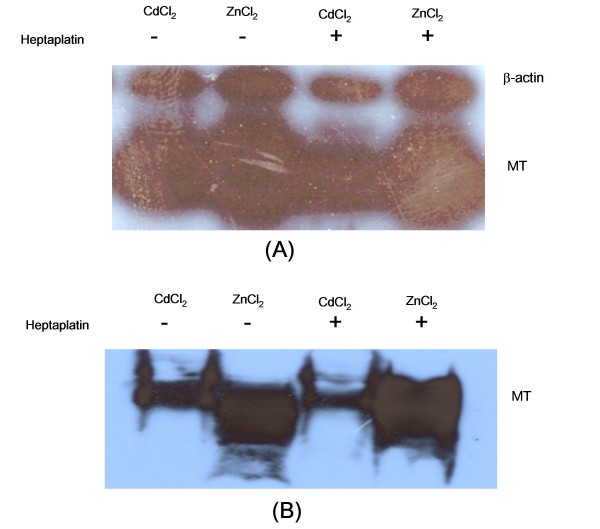
Effect of heptaplatin on MT induction by heavy metals in the SNU-601 cell lines. The SNU-601 cells were treated with 32 μM CdCl_2 _or 200 μM ZnCl_2 _in the presence or absence of 200 ng/ml heptaplatin for 24 hr. The MT expression level was determined by (A) the RT-PCR assay and (B) the Western blotting method.

After a 24-h treatment, MT expression level was determined by the RT-PCR assay and Western blot analysis. Fig. 9 shows that heptaplatin inhibits the MT gene expression induced by cadmium but not zinc, suggesting the differential modulation of metal-induced MT expression.

## Discussion

The cisplatin analogs are among the most active and widely used cytotoxic anticancer drugs. However, the acquisition or presence of resistance significantly undermined the curative potential of these drugs against many malignancies. Alterations in the cellular pharmacology, including decreased drug accumulation, increased cellular thiol levels and increased repair of platinum-DNA damage, have been observed in the cisplatin-resistant cancer cell lines [19]. Since heptaplatin is effective against cisplatin-resistant cancer cells, its development has shed light on cisplatin-refractory cancer patients. However the mechanism by which heptaplatin is effective against cisplatin-resistant cancer cells is unclear. Since MT is involved in cisplatin resistance [26], the molecular mechanisms for the effect of heptaplatin against the cisplatin-resistant cancer cell lines was investigated with respect to the involvement of MT. In this study, the gastric cancer cell lines expressed different basal MT mRNA levels, whose mechanisms remain to be determined. It was suggested that DNA hypomethylation was responsible for the higher basal hMT-IIa mRNA levels in the cisplatin-resistant cells [20]. In this study, MT expression in the gastric cancer cell lines was not always related to the resistance to cisplatin, suggesting the involvement of other mechanisms except MT. But heptaplatin is more effective against the MT-overexpressing gastric cancer cell line selected for resistance to cisplatin than cisplatin and carboplatin were. In addition, the cytotoxicity by cisplatin and carboplatin but not heptaplatin was reduced by a pretreatment with zinc, which can induce MT remarkably (data not shown). Taken together, heptaplatin was more effective against both cisplatin-resistant gastric cancer cell subline SNU-601/CIS and MT-overexpressing SNU-638 than was cisplatin or carboplatin. These results suggest a possibility that the cytotoxicity of heptaplatin was less influenced by MT. To test the hypothesis, the function of MT involved in the cytotoxicity of the cisplatin analogs was confirmed by the transfection of MT. The cytotoxicity of heptaplatin was not influenced by the MT transfection, which indicates that the anticancer activity of heptaplatin to the cisplatin-resistant gastric cancer sublines could be less associated with MT. In addition, heptaplatin inhibited the MT expression caused by cadmiun but not zinc. This result suggests that metals differentially regulate MT, which is supported by the literature. The inhibition mechanism for H7, a protein kinase C inhibitor, may be different between the zinc- and cadmium-treated cells. H7 blocks zinc transport but not cadmium transport although rottlerin, a PKC inhibitor that can block both cadmium and zinc transport [21,22]. Hypoxia activates MT expression through the metal response elements and that this activation involves the metal transcription factor-1 (MTF-1) [23]. MTF-1 was found to be present in the cytoplasm of the cells. Upon zinc stimulation, MTF-1 was translocated to the nuclei and activated MT gene expression [24]. However, no MTF-1 translocation was observed in cells treated with cadmium [25]. Whether or not MTF-1 is involved in inhibiting the cadmium-induced MT induction by heptaplatin remains to be determined.

In summary, these results indicate that molecular mechanisms responsible for the effect of heptaplatin against the cisplatin-resistant gastric cancer sublines is at least in part due to the lower involvement of MT as well as its attenuation of MT induction.

## Materials and Methods

### Cell culture and the selection of the gastric cancer cell subline for cisplatin resistance

The human gastric cancer cell lines (SNU-1, SNU-5, SNU-16, SNU-484, SNU-601, SNU-620, SNU-638, and SNU-668) were obtained from the Cancer Research Center in Seoul National University (South Korea). The cells were cultured in RPMI 1640 (GibcoBRL Grand Island, NY, U.S.A.) supplemented with 10% FBS (Sigma Chemical Co. St. Louis, MO, U.S.A.). The cells were maintained as a monolayer culture and subcultured at confluence. The cisplatin-resistant gastric cancer cell subline was selected by chronic exposure to gradually increasing cisplatin concentrations ranging from 200 ng/ml (IC_50 _value) to 2,000 ng/ml on an intermittent dosage schedule.

### Cytotoxicity assay

The *in vitro *cytotoxicity of the drugs was determined using an MTT (Sigma Chemical Co. St. Louis, MO, USA) assay previously described by Pieters *et al*. [26]. The 50% inhibitory concentration (IC_50_) for a particular agent was defined as the drug concentration that causes in a 50% reduction in the number of cells compared to the untreated control for 3 days. The IC_50 _values were determined directly from the semilogarithmic dose-response curves. Cisplatin and carboplatin were obtained from Sigma Chemical Co. (St. Louis, MO, USA) and heptaplatin (Sunpla^®^) was generously donated by SK Pharmaceutical (Seoul, Korea). Cisplatin analogs were dissolved in phosphate buffered saline and used immediately because of their instability in aqueous solution.

### RNA extraction RT-PCR assay

The total RNA was extracted from the cells using the acid guanidium thiocyanate-phenol-chloroform method [27]. The MT and β-actin mRNA transcripts were detected using an RT-PCR assay. The RNA from each sample was reverse transcribed using 200 units of Moloney murine leukemia virus reverse transcriptase (GibcoBRL, Grand Island, NY, U.S.A) and oligo (dT) primer for 1 h at 37°C. The resulting cDNA was diluted 1:5 with distilled water and amplified with 2.5 units of Taq polymerase (Perkin-Elmer, Foster City, CA, USA) and 10 pmole of each primer using a GeneAmp PCR2400 (Perkin-Elmer, Foster City, CA, USA). The MT expression level was detected with the sense and antisense primers corresponding to the nucleotides (5'-ATGGACCCCAACTGCTCG) and (5'-TCAGGCGCAGCAGCTGCA), respectively, of the published cDNA sequence [28] and yielded a 220-bp PCR product. The β-actin expression level, as a control of the RNA quantity, was detected with the sense and antisense primers corresponding to the nucleotides 1912–1932 (5'-GACTATGACTTAGTTGCGTTA) and 2412–2392 (5'-GCCTTCATACATCTCAAGTTG), respectively, of the published cDNA sequence [29], yielding a 501-bp PCR product. Twenty one PCR cycles for MT was carried out as follows: denaturation at 94°C for 30 s, annealing at 57°C for 60 s, and extension at 72°C for 1 min was used. Sixteen cycles of PCR for β-actin of denaturation at 95°C for 30 s, annealing at 53°C for 30 s, and extension at 72°C for 30 s. After the last cycle, all the PCR products were subjected to a final extension for 5 min at 72°C. For quantitation, 5 mCi/ml of [α-^32^P] dCTP was added to each reaction mixture. Subsequently, the PCR products were combined and electrophoresed on 7.5% nondenaturing polyacrylamide gels. The bands were scanned using a densitometer (Pdi, Huntington Station, NY, USA). The quantity of each mRNA transcript was normalized with that of β-actin mRNA. Autoradiographic films of the RT-PCR assay were subjected to densitometric analysis using a densitometer.

### Protein extraction and Western blot analysis for MT

The total cell lysates from 2 × 10^6 ^cells were prepared by lysing the harvested cells in an extraction buffer (1% NP-40, 0.5% sodium deoxycholate, 0.1% SDS in phosphate-buffered saline) supplemented with 2 mM phenylmethylsulfonyl fluoride (Sigma Chemical Co. St. Louis, MO, USA) and 10 μg/ml leupeptin (Sigma Chemical Co. St. Louis, MO, USA). The DNA was sheared by sonication. The sample buffer for MT analysis was used without 2-mercaptoethanol [30]. Western blotting analysis was performed using a slight modification of the method reported by Towbin *et al*. [31]. The proteins were transferred onto a nitrocellulose membrane by electroblotting at a current of 60 V overnight using a transfer buffer containing 5% mercaptoethanol. The membrane was incubated in a blocking solution (5% skim milk) for 1 hr at room temperature, washed, and then incubated with a monoclonal mouse antibody (E9 clone, 1:500, Dako Corporation, Carpinteria. CA, USA) for MT-I and MT-II. The membrane was washed and incubated with horseradish peroxidase-conjugated secondary antibodies (diluted 1:1000) for 1 hr. The membrane was then stained using the ECL detection kit (Amersham Biosciences Corp., Piscataway, NJ, USA).

### Production of stable transfectant cell line

Mammalian transfectants were produced as reported previously [18]. The mammalian expression vector, pIRESneo2 (Clonetech, Palo Alto, CA, USA), was used to clone the MT. A 220 base pair cDNA fragment containing the full open reading frame of human MT was amplified by RT-PCR from the RNA of AML-2/PQ400 [17], a paraquat-resistant AML-2 cell line that highly over-expresses MT. The PCR primer pairs were as follows: the forward primer 5'-CGGGATCCATGGACCCCAACTGCTCG-3' to introduce a *Bam*H I site as underlined, and reverse primer: 5'ATAAGAATGCGGCCGCTCAGGCGCAGCAGCTGCA-3' to introduce a *Not *I site as underlined. The resulting MT PCR product was cloned to a TOPO TA cloning kit (Invitrogen, Carlsbad, CA, USA). After confirmation of the sequencing, the MT was cut from the TOPO plasmid vector (pCR 2.1-TOPO), purified, subcloning to the multicloning site of the expression vector pIRESneo2 and subsequently sequenced to confirm the correct open reading frame. Transfection was performed using the LipofectAmine Plus reagents (GIBCO BRL, Grand Island, NY, USA). Approximately 1 × 10^6 ^of the SNU-601 cells were plated into a 60 mm tissue culture dish and cultured overnight. Prior to transfection, the growth medium was replaced with the serum free RPMI 1640 media and cultured. The LipofectAmine reagent containing 2 μg of pIRES/Mock and pIRES/MT was combined with the Plus reagent and applied to the cells. After culturing for five hours, the media was replaced with RPMI 1640 containing 10% FBS. After selection by culture in the medium containing 0.2 mg/ml of G-418 (GIBCO BRL, Grand Island, NY, USA), the emerging colonies were isolated with cloning rings. The stable transfectants, SNU-601/Mock and SNU-601/MT, were obtained. The selection medium was changed every 2–3 days.

### Determination of reactive oxygen species (ROS) using a fluorometric probe

Dichlorofluorescin (DCFH) was used to measure the ROS concentration. After 2', 7'-dichlorofluorescin diacetate (DCFH-DA) crosses the membrane, it is de-esterified to DCFH which is oxidized to fluorescent DCF by the ROS [32]. Phosphate-buffered saline containing 1 × 10^5^/ml SNU-601/Mock or the SNU-601/MT cells was incubated with 1 μM DCFH-DA at 37°C for 4 hr. After incubation, the DCF fluorescence intensity was determined using a fluorometer at 485 nm for excitation and 530 nm for emission.
